# An Electro-Oculogram Based Vision System for Grasp Assistive Devices—A Proof of Concept Study

**DOI:** 10.3390/s21134515

**Published:** 2021-07-01

**Authors:** Rinku Roy, Manjunatha Mahadevappa, Kianoush Nazarpour

**Affiliations:** 1 Advanced Technology and Development Centre, Indian Institute of Technology, Kharagpur 721302, India; 2 Indian Institute of Technology, School of Medical Science and Technology, Kharagpur 721302, India; mmaha2@smst.iitkgp.ac.in; 3 Edinburgh Neuroprosthetics Laboratory, The University of Edinburgh, Edinburgh EH8 9AB, UK; kianoush.nazarpour@ed.ac.uk

**Keywords:** electrooculogram, artificial vision, inexpensive imaging, grasp assistive device, brain–computer interface

## Abstract

Humans typically fixate on objects before moving their arm to grasp the object. Patients with ALS disorder can also select the object with their intact eye movement, but are unable to move their limb due to the loss of voluntary muscle control. Though several research works have already achieved success in generating the correct grasp type from their brain measurement, we are still searching for fine controll over an object with a grasp assistive device (orthosis/exoskeleton/robotic arm). Object orientation and object width are two important parameters for controlling the wrist angle and the grasp aperture of the assistive device to replicate a human-like stable grasp. Vision systems are already evolved to measure the geometrical attributes of the object to control the grasp with a prosthetic hand. However, most of the existing vision systems are integrated with electromyography and require some amount of voluntary muscle movement to control the vision system. Due to that reason, those systems are not beneficial for the users with brain-controlled assistive devices. Here, we implemented a vision system which can be controlled through the human gaze. We measured the vertical and horizontal electrooculogram signals and controlled the pan and tilt of a cap-mounted webcam to keep the object of interest in focus and at the centre of the picture. A simple ‘signature’ extraction procedure was also utilized to reduce the algorithmic complexity and system storage capacity. The developed device has been tested with ten healthy participants. We approximated the object orientation and the size of the object and determined an appropriate wrist orientation angle and the grasp aperture size within 22 ms. The combined accuracy exceeded 75%. The integration of the proposed system with the brain-controlled grasp assistive device and increasing the number of grasps can offer more natural manoeuvring in grasp for ALS patients.

## 1. Introduction

Prior to initializing the hand movement for grasping the target object, we first visually assess the object to determine its shape, size, and orientation [1,2] which will be further used for manipulating the grasp type, grasp aperture and wrist orientation accordingly. The sensory information is then processed by the Central Nervous System (CNS) for motor planning in the cortex and implementation using the muscular system [3,4]. On reaching the object, the wrist and fingers of the hand move accordingly to hold the object; the gaze fixates on the target until the completion of the whole task [5]. People with Amyotrophic Lateral Sclerosis (ALS), also known as Lou Gehrig’s disease, lose almost all their voluntary muscle control, including the upper limb movement [6]. Most of them uses a grasp assistive device for carrying out their daily activities. For controlling the grasp type in those assistive devices, various invasive and non-invasive techniques, e.g., ECoG, EEG, etc. References [7,8,9] has already been used. However, the grasp aperture and orientation are still not possible to identify from the brain response alone. However, due to the high amount of GABAergic transmission in oculomotor nuclei [10]. Those people can recognize the target object and its shape and size with the visual assessment but not able to convey that information to the assistive device for actual grasp implementation. An external vision system is required in addition to the grasp assistive device for those patients to identify and process that information to achieve a stable grasp with the grasp assistive devices. In this paper, we have proposed a vision system that can be integrated with the brain-controlled grasp assistive device for the use of ALS patients to restore their grasp activities in future.

For myoelectrical-controlled prosthetic limb, various vision-augmented systems have already been proposed for identifying the object shape and size in terms of estimating the grasp type and aperture [11,12,13,14,15,16]. Dǒsen et al. [17,18] developed a dexterous hand with an integrated vision based control system. The user controlled the prosthesis hand and the activation of the camera with myoelectric signals. With a simple object size detection method and the measurement of distance, they achieved an accuracy of 84% in grasp type selection. However, due to the placement of webcam on the prosthesis itself, the prosthetics were required to be brought in proximity of the target object to function correctly. Markovic et al. [12,14] overcame this problem using an augmented reality (AR) glasses, developed a rule-based model to provide faster object shape and size by stereo-vision associated with artificial proprioceptive feedback for grip aperture size. However, their vision system required the user to look straight at the target object by adjusting their heads. Hence, for locating the target object most of these existing vision system required some amount of voluntary muscle activities [19] which are restricted for most of the ALS patients.

Along with the control approach, the use of computer vision systems entails a real-time implementation of sophisticated algorithms typically [16,20,21]. With the advent of deep learning methods, grasp research has considerably advanced. Lenz et al. [22] demonstrated a two-step deep learning model to identify the suitable grasping spot of the target object based on its size, position, and orientation. Kopicki et al. [23] developed a one-shot learning model to select suitable grasp for different objects. In another approach, Ghazaei et al. [15,16] classified simple household objects into various grasp classes with a convolutional neural network (CNN). However, the model was not able to provide a satisfactory result for identifying the novel objects. In a recent study [24], a more advance multi-modal CNN was trained with both Depth and RGB information to recognize the novel objects belongs to four different grasp types (cylindrical, spherical, tripod, and lateral). However, all those systems require a GPU to train the network. Along with that, a large object-image database is needed for the training procedure. To avoid the use of GPU in the portable vision system, we used openCV Deep neural Network (DNN) module in our recent study [25]. However, the requirement of large object-image database is still there for training.

For locating the target object, in this paper, we utilized the human gaze movement which is unimpaired for the targeted patients. Scientists have already tried two approaches to measure EEG and eye Movement simultaneously [26]. In the first approach, EOG was recorded with EEG to detect eye movements [27], whereas in the second approach eye movement was recognized through an eye tracker [26]. Though the last approach is providing better accuracy, the wearable EOG goggles are preferred more for Human–Computer Interaction (HCI) purposes [28]. In this paper, we used the EOG signals to control the pan and tilt of a single webcam that was mounted on a cap to localize the object of interest. Recently and as a proof of principle, we demonstrated the combined use of an EOG controlled webcam and a deep learning network for object identification [29]; however, the device was not used in a cluttered environment. Along with this object localization approach through EOG, we also designed a simple image processing pipeline to reduce the involvement of high processor and storage capacity to execute a single task. The boundary of the targeted object was used as the ‘signature’ of the object to determine the object size and wrist orientation. We assumed that the grasp type can be identified from the brain measurements. As most of the house-hold objects lie in the ‘palmar grasp’ type [30], we catered the objects of this category for our experiment. Based on the wrist orientation, we classify objects in two classes: Palmar Wrist-Neutral and Palmar Wrist-Pronated. We tested the device with able-bodied participants in real-time.

## 2. Methods

**Figure 1:**
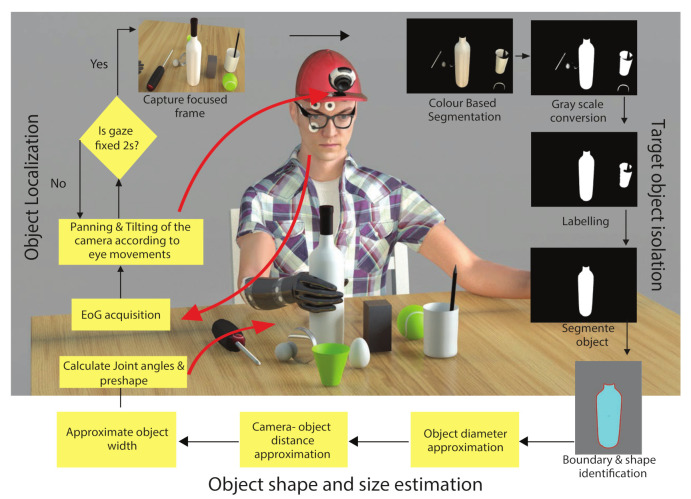
The schematic process flow diagram of the EOG-driven computer vision system for grasp control. The grasp assistive device is illustrated here with a black sleeve. (This is a schematic of the overall experimental process. In this study, we did not test the developed vision system with any assistive devices).

Figure 1 shows the schematic of the proposed vision system. The system comprises three main parts: (1) Object localization using the EOG signals (Section 2.2); and (2) Grasp orientation and aperture estimation using image processing (Section 2.3); and finally (3) Grasp actuation. This paper addresses the first two.

### 2.1. Participants

Fifteen volunteers (age: 27–32 years) took part in this experiment. All were able-bodied, right-handed and free from any neurological or motor disorders. The study was conducted according to the guidelines of the Declaration of Helsinki, and approved by the local ethics committee at Indian Institute of Technology, Kharagpur. All participants gave informed written consent. Five volunteers participated in an initial system calibration experiment. The other ten participants tested the complete system.

### 2.2. General Setup, Calibration and Object Localization

**Figure 2:**
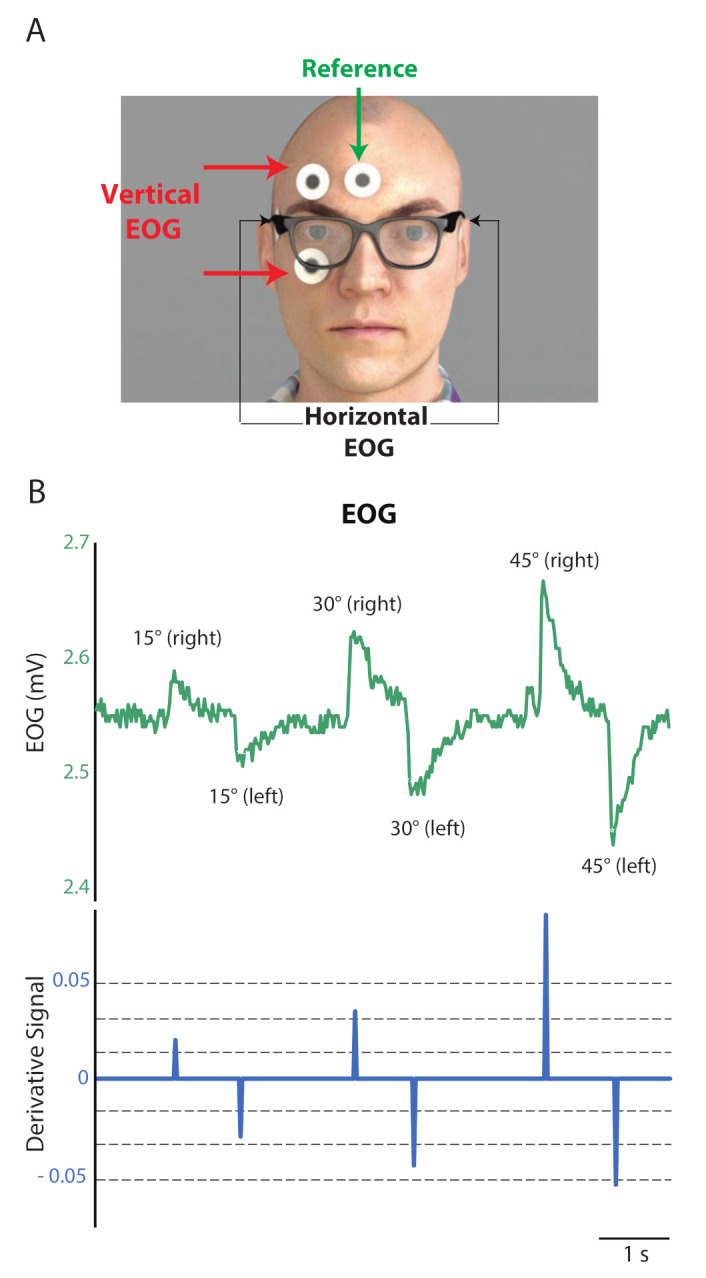
(**A**) EOG and reference electrode placement; (**B**) Example raw EOG signal (**top**) and the extracted eye movement signal with differentiation and thresholding (**bottom**).

- EOG acquisition and analysis:

To measure the electrical activity, we used standard Ag/AgCl disposable electrodes. We placed a reference electrode on the forehead, as shown in Figure 2A. The acquired EOG signals were amplified (gain: 2000) and filtered (2–16 Hz, 8th order butterworth filter) to generate the control signal. An ATmega328P micro-controller converted the conditioned EOG signals into digital and transmitted them to a PC via serial communication. In software (MATLAB^®^), we differentiated the horizontal and vertical EOG signals to detect the movement of the eyes. To remove small fluctuations from the differentiated signal, we applied a threshold to both differentiated EOG channels. Figure 2B (top) illustrates an example of the EOG signal. The corresponding differentiated and thresholded signal is shown in Figure 2B (bottom). We mapped the extracted peaks to gaze angles.

**Figure 3:**
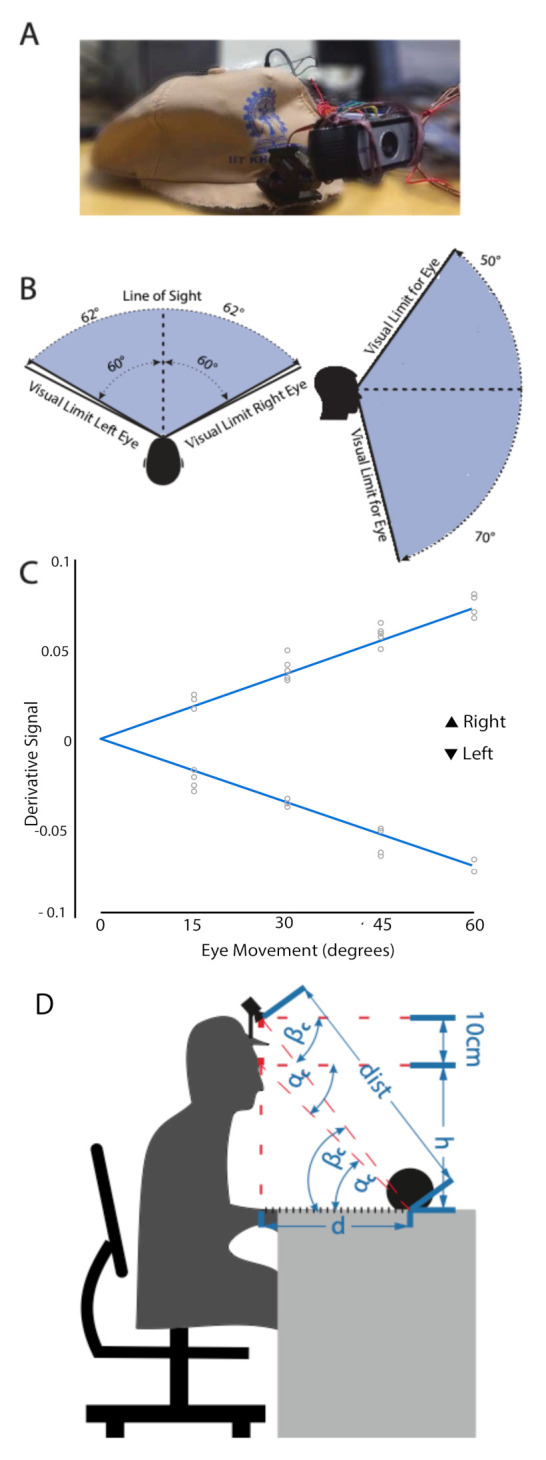
(**A**) The instrumented cap visor; (**B**) The users’ field of vision in the horizontal and vertical planes; (**C**) Interpolated line for estimating gaze angles; (**D**) Camera tilting angle $\beta_{c}$ estimation for an object placed at distance *d*, on the tabletop *h* below the eye level.

- Device description:

Figure 3A shows the webcam, mounted on the cap. Two small servo motors, $S_{P}$ and $S_{T}$, controlled the panning and the tilting of the camera with the corresponding horizontal and vertical EOG peaks, $P_{H}$ and $P_{V}$. We set the servos initially at 0${}^{\circ }$ vertically and horizontally. They covered the horizontal and vertical gaze ranges fully (Figure 3B). We streamed the output of the webcam into a PC with Intel^®^ Core™ i3 processor that worked with Windows™ 7 64-bit OS. The Image Acquisition Toolbox™of MATLAB^®^ captured the video stream.

- Calibration of gaze angles:

In the calibration experiment, the EOG amplitudes for the various gaze angles were recorded from the five participants. Participants sat in front of a table of a semi-spherical arrangement of a few graduated scaling points horizontally and vertically placed. The radius of the reach space was 50 cm, as per the range of human arm length. The horizontal plane comprised nine target points at 15${}^{\circ }$ intervals. Five equidistant targets spanned the 60${}^{\circ }$ vertical plane above and below the eye. The centre target points for both the planes were coincident with each other and acted as a reference point. The chosen angular limits complied with the lateral and vertical range of human field of vision: $\pm 62^{\circ }$ and $\left[-70^{\circ },50^{\circ }\right]$, respectively, as illustrated in Figure 3B. Participants moved their eyes to each of the horizontal and vertical targets. Between every two successive targets, the participants returned their gaze to the initial reference point. The detected EOG peaks from the five participants for the selected target points are demonstrated in Figure 3C which displays a linear relationship between the Left-Right gaze angles and the processed EOG signal peaks. A similar relationship was obtained for Up-Down directions. This relationship was further used to find out the gaze angle from the detected EOG amplitude value.

- Device workflow:

We input the calculated horizontal gaze angle (θ) directly to servo $S_{P}$ as a webcam panning angle ($\theta_{c}$). For the camera tilting angle ($\alpha_{c}$), we adjusted for the 10 cm difference between the eye level and camera, as shown in Figure 3D. The re-calibration of camera tilting angle to $\beta_{c}$, Equation (1), allows the webcam to intersect with the eye gaze line at the target object plane. If the distance between the eye level and the tabletop is *h* cm and an object placed at distance *d* cm from the user, then the tilting angle $\beta_{c}$ is

(1) $$\beta_{c}=\tan^{-1}\frac{h+10}{d}=\tan^{-1}\frac{h+10}{h\cot\alpha_{c}}.$$

Considering the human hand reaching range, we set the upper limit of *d* to 40 cm. Furthermore, we can calculate the distance between the camera and the target object (dist) with

(2) $$dist=\left(h+10\right)/\sin\beta_{c}.$$

During object localization procedure, two servo motors which are responsible for camera panning and tilting were initialized at 0${}^{\circ }$. The webcam was attached with the PC through the serial port and accessed through the MATLAB interface. The camera was fixed parallel with the horizontal eye level. The centre position of the camera was adjusted in the same vertical line of reference electrode (middle of the forehead). As soon as the user move their eyes towards the target object, eye gaze angle as well as movement direction starts changing and camera starts to pan and tilt accordingly the detected EOG peak amplitude. When the participant fixates on the object, there will be no further movement of eyeballs. As a result, the EOG signal becomes steady with no prominent peaks, both the servos stopped at the desired angle. If the system does not recognize any further peaks for more than 2 s, the camera captures a snapshot of the focused region. The target object assumed to be lie in the centre as the user focus on the target during object localization. We configured the camera to acquire the image of 572×812 pixels resolution in the RGB mode. Algorithm 1 describes the object localization process.
**Algorithm 1:** Object Localization
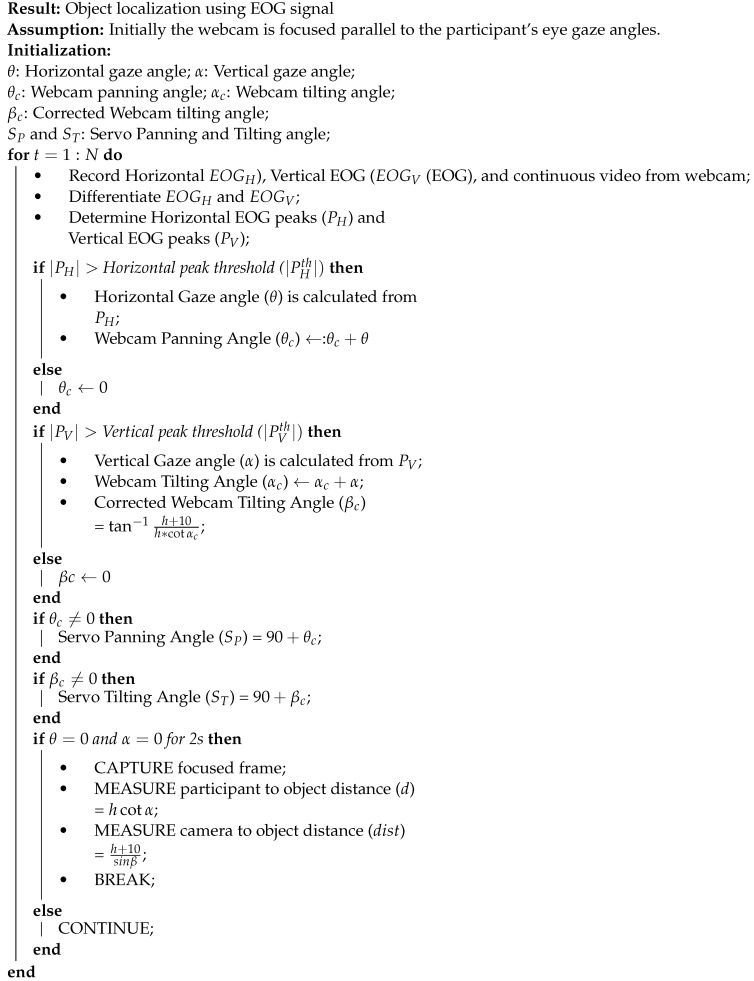



### 2.3. Grasp Orientation and Aperture Estimation

**Figure 4:**
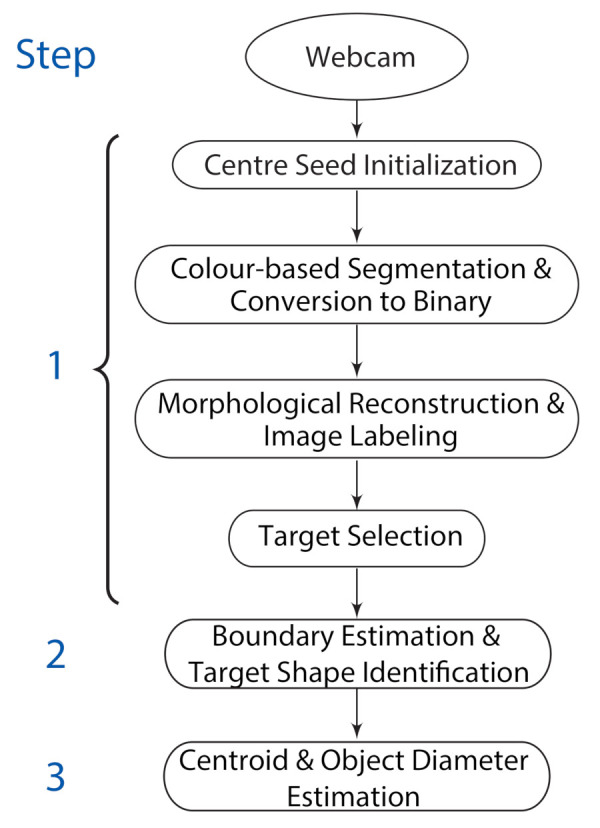
The image analysis process flow comprising (1) Target object isolation; (2) The signature model extraction; and (3) Object centroid and diameter determination.

The captured image was used to identify the grasp orientation and the estimation of grasp aperture. Grasp orientation depends on object shape whereas grasp aperture depend on object size. The image analysis comprised three main parts: (1) target object isolation, (2) object signature extraction, and (3) object centroid and diameter determination. Figure 4 depicts the process.

We assumed that our desired object was in the focus of the camera; so it was lying in the centre position of the captured frame. Due to this reason, we have isolated the middle object of the frame as the object of interest. To segment it, the central pixel of the image was selected as the seed. A 7 × 7 neighbourhood pixel window was taken around the seed point. We used this window to segment all portions in the image containing the same colour as the seed point. All closed contours obtained in the previous step were considered to belong to the object. We assigned the pixels inside the contours to 1 and other pixels to 0, before dilation to connect the nearest pixels. The segmented area from the above step may contain both the target and other background segments. To isolate the target under these conditions, we labelled all segmented areas. We considered the area containing the same label as the central pixel as the target. We discarded all other segmented areas.

**Figure 5:**
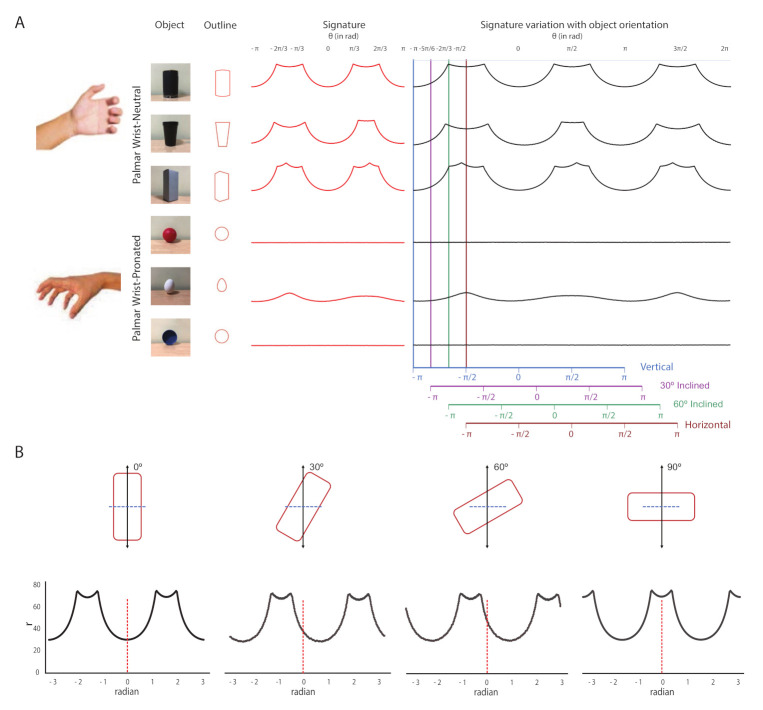
(**A**) Signature of different objects and their variation with orientation and (**B**) An example of the relationship between the boundary signature and the orientation of the object.

Wrist orientation during any grasp depends on the inclined angle of the objects. When any object rotates in its focal plane, the boundary of the whole object remain same, only the angle between the central horizontal axis of the object with the y-axis varies according to the object orientation (refer Figure 5B above). For this reason, we utilized the Boundary of the targeted object to estimate the object orientation. Here, we developed a ‘Signature’ model approach to classify object contours. Boundary values, as the signature of the particular object, of each segmented area were calculated from the image. We utilized the polar coordinates of the boundary values to classify signatures. As a proof of principle, our models only catered for the objects that can be held with Palmar Wrist-Neutral and Palmar Wrist-Pronated condition. Example signature of different object shapes with the mentioned grasp patterns are shown in Figure 5A. In this figure, we plotted the example signatures as polar angles versus radius. The Figure exhibits the variation of boundary profile with an object inclination angle. Four example orientations in [−π,π] are shown to highlight the effect of orientation on the signature curve. The figure shows that the objects requiring Palmar Wrist-Neutral grasp have more than 2 maxima irrespective of their orientation, whereas the objects normally hold with Palmar Wrist-Pronated condition having either no maxima or fewer than 2 maxima. The maxima were developed due to the existence of the corners in the mentioned object. As we rotate the object in its focal plane, the corner positions of the object shifted. As a result, peak positions in the Signature profile also varies. This phenomenon is further demonstrated in Figure 5B as isolated plots for the shift of the object signature profile according to the object orientation. The figure depicts that the distance of the first peak in the graph from the origin can be used to identify the orientation of the object. It can be observed that only the signature curve shifts with the orientation, keeping the overall nature of the plot. The objects usually hold with Palmar Wrist-Pronated condition contain either no or very few numbers of corners in the objects. For this reason, the Signature model of those objects contains either zero or less than two maxima (refer Figure 5A). During each experimental trial, the signature of the segmented object was estimated and then the number of peaks in the Signature graph were identified. For the objects with Palmar-wrist neutral type, the distance of the first peak from the origin was also estimated to check the orientation of the objects.

Along with the object orientation, object width also need to measured to identify the grasp aperture. Humans normally grasp an object at its centroid to avoid slippage [31,32]. Therefore, we estimated the object width along the centroid to determine the grasp aperture size. We converted the width to a real unit with $W=w_{p}\frac{dist}{f}$ where *W* and $w_{p}$ are the widths of the object in cm and in pixels, respectively. The constant f=428 is the focal length of the webcam.

### 2.4. Whole System Testing

**Figure 6:**
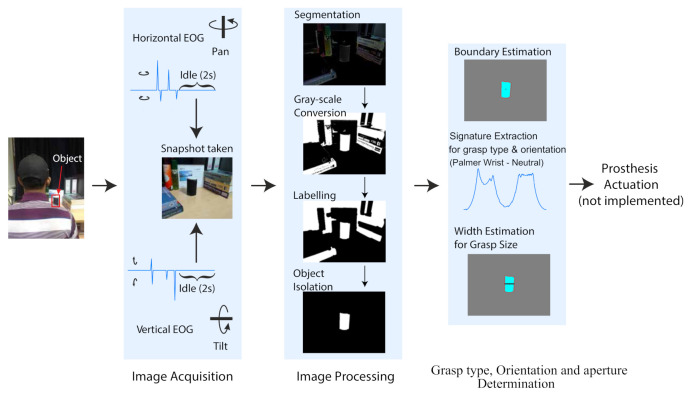
A representative example of correct object detection, signature extraction and width estimation.

Participants (n=10) sat comfortably in an armchair, wearing the instrumented cap at a table that was cluttered with various office objects, as in Figure 6. We placed a single object of interest in each trial in this cluttered environment. The objects were (i) a can (dia: 6.6 cm), (ii) a plastic cup (dia: 6.4 cm), (iii) a rectangular shaped box (dia: 5.4 cm), (iv) a ball (dia: 2.8 cm), (v) an egg (dia: 3.8 cm), and (vi) a lid of a jar (dia: 6.3 cm). We categorized these objects into two grasp patterns based on their normal holding posture—Palmar Wrist-Neutral and Palmar Wrist-Pronated. In most of the trials, objects were placed in their regular orientations. In 20% of trials, we changed the orientation randomly to identify the angle of inclination from their signature. The range of object diameters (2.8–6.6 cm) was chosen to test the system efficiency in identifying the correct object width. Each participant performed a total of 30 trials; 5 trials for each object. In each trial, the selected object was put on the table at a random position within a distance (*d*) between 15 and 40 cm from the participant. Before starting each trial, the participant was requested to stay stable in their position as much as possible during the experiment. The height between their eye-level to table-top distance (*h*) was measured in this fix position using a measuring tape.

## 3. Results

Figure 6 shows a representative trial including the recorded EOG signal, the acquired image in the cluttered scene, and the results of Grasp orientation identification and object width estimation. In this figure, the participant moved their eyes towards the object of interest. The camera followed the movement of the eye and captured a frame when the participant fixed their eye gaze on the object for 2 s. In this trial, a black cylindrical object of diameter 6.6 cm was placed on the table on the right side of the participant. The implemented algorithms correctly extracted the signature of the object and identified the wrist orientation as Neutral condition. It also identified the object orientation from the same plot by observing the position of the first peak. Width measurement was also performed accurately. In a real-time implementation with a grasp assistive device, these decisions would be transferred to the device and the control will be returned to the user who controls the device with their brain signals. The video is in the supplementary material.

**Figure 7:**
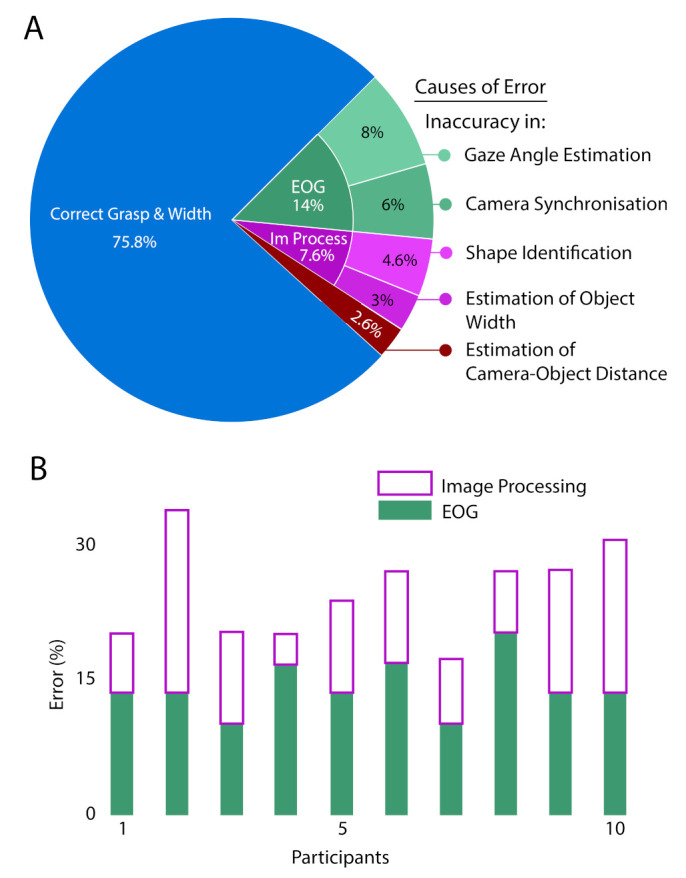
(**A**) Overall, the proposed system achieved an accuracy of 75.8% in selecting the object and recognizing the correct grasp orientation and size. In 14% of trials, the position of the camera caused the misinterpretation of the EOG peak. In 7.6% of trials, an error occurred in the image processing unit. In 2.6% of the trials, an error occurred due to inaccurate distance calculation; (**B**) Distribution of errors in decision making with respect to individual participants.

- System accuracy and Error Analysis:

Each object used in this experiment has a definite shape and width; the orientation of the objects was also varied randomly during the trials. The identified grasp orientation in each trial, also the estimated object width was compared with the defined object parameters to measure the overall system accuracy. In this study, the developed assembly had an overall accuracy of 75.8% (a total of 300 trials). We listed all the specific reasons for incorrect identification. There were three main caused for inaccuracy in (1) EOG analysis (14%); (2) image processing (7.6%); and (3) estimation of the distance between the camera and the object (2.6%), as shown in Figure 7.

In 42 cases (14%), the error occurred in locating the correct target object. The main reason behind this error was the mismatch of recorded EOG amplitude with the selected threshold ranges. As a result, the system failed to detect the correct eye gaze angle from the recorded EOG peaks. Due to this condition, the developed vision system was unable to move in the desired direction for locating the object of interest and resulted in inaccurate or no target identification (8% cases). Another reason behind this incorrect object localization was taken place due to the rapid movement of the participant’s eyeball. To process the EOG signal and to identify the gaze angle, the system needs a 1ms gap between two successive eye movements; otherwise, the system failed to detect the correct gaze angle. This resulted in the camera panning and tilting angles to not be in synchrony with the change of eye gaze angle. In 6% of trials, object localization was incorrect due to that reason.

Once the system identified the correct object, the snapshot was processed through several image processing pipelines to segment out the object from the cluttered scene, to identify the wrist orientation along with object width estimation. Errors can also occur during this image processing phase. Here, we extracted the target object from the cluttered scene using a color-based segmentation method. Sometimes, the algorithm accumulated the same coloured nearby segments of the image as a part of the target object; as a result, the incorrect boundary was estimated for the identified objects. In our study, we faced this problem in only 4.6% cases of 300 trials; so, we did not achieve correct Grasp-Orientation in those cases due to their wrong signatures. Even though segmenting out the correct object from the frame, the system can fail to estimate the correct object width from it. As we estimated the real object-width from the image object-width (in terms of the pixel), incorrect width estimation in the image also produced the wrong width measurement. If our measured object-width lies between ±15% of their actual width, then we have considered it as a successful attempt. This error can be occurred mainly due to two reasons. In the captured frame, 3D objects were projected on a 2D plane. Therefore, some portions of the object edges were clipped during the segmentation stage. As a result, the object width estimated from the image in terms of pixel got changed. In 3% of the trials, this type of error occurred, and incorrect object-width was estimated for this reason. In spite of having a correct object width in terms of the pixel, we observed a small error of 2.6% in estimating real object-width due to the inaccurate camera-object distance approximation due to discrepancy of *h* between the initial (before starting of the experiment) and the final position (the moment camera capture the frame) of the participant.

Figure 7 shows the distribution of errors. In addition, we categorized the errors according to the origin, namely acquisition or analysis of the EOG signals or image processing. Due to a minimal number, we ignored the error in object-width estimation for the wrong dist calculation (2.6%). In this figure, each bar represents the percentage of failed trials for each participant. The figure demonstrates that the system was unable to identify the correct object with EOG in less than 15% (avg.) of trials of each participant; whereas, it failed to identify the grasp pattern and object-width from image processing in less than 10% (avg.) of trials. Results show that despite our subject-specific calibration (*h*), the errors between participants were comparable.

**Figure 8:**
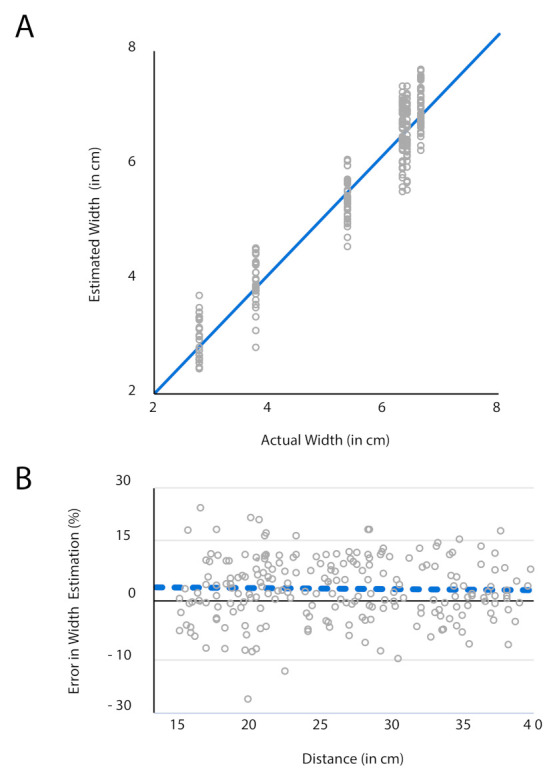
(**A**) Discrepancy between calculated and actual width; (**B**) Estimation error (%) versus the distance between the participant and the target object.

In our experiment, we used the objects in the width range of 2.8–6.6 cm. We observed that the rate of failure is high in locating smaller objects (ball and egg with diameter 2.8 and 3.8 accordingly) than larger. Among the 100 trials with the smaller objects, we achieved success in only 42 cases. Incorrect gaze angle estimation for a very small variation of eye-movement is one of the reasons behind this issue. Besides that, segmenting the smaller objects can also cause an error due to intrusion of other larger objects nearby. However, we expected that size estimation for the larger objects would be more accurate than that for the smaller objects. Agreeing with the predictions, results in Figure 8A show that the error in estimation remains approximately ±15% of the actual width when the object size increases.

The accuracy of any vision system also depends on the object identification and object-width determination ability of the system from various distances. We, therefore, varied the distance of the object randomly for each trial, within 15–40 cm from the participant. Error in the estimated object-width in those random distances (measured with tape) is shown in Figure 8B. The figure illustrates that error in the calculation of the width of the target object was not correlated with the participant-to-object distance. It is also displayed that in most of the trials, object-width was over-estimated than its original. A blue dashed line which is the mean of all the error in width (%), indicated this over-estimation. This over-estimation does not impair real-life control of any grasp assistive device. It allows the user to adjust better the assistive device around the object before actuation. On average, the proposed system over-estimated the width by about only 3% (blue dashed line).

**Figure 9:**
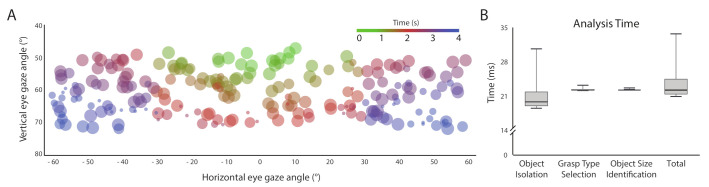
(**A**) Distribution of time required to localize an object and determine the width of the object; (**B**) The required time for different stages of image processing.

- System responsiveness:

Figure 9A illustrates the time required for capturing the correct object image as a color-scaled bubble plot. The size of the bubble depicts the relative width of the objects, whereas the color of a bubble indicated the total time taken to move the eyes horizontally and vertically to fixate on the object of interest. The figure shows that most of the green-ish bubbles lie within the horizontal viewing angles and blue circles in the extremities. When an object was placed within a participant’s close viewing angle (±10${}^{\circ }$), the time required was less. However, when it was kept near the extremities of the human viewing angle (>±30${}^{\circ }$), participants took more time in finding the correct object. Moreover, smaller objects placed closer to the participants required higher object localization time. Figure 9B shows that the whole image processing algorithm steps and grasp type and size estimation stages take about 22 ms, on average. It is not surprising that the image processing steps, namely, object isolation and grasp type selection, take a large 20 ms portion of total 22 ms. The system does not take any extra time for orientation detection as it is recognized from the same signature used for grasp type selection.

## 4. Discussion

In this study, we implemented an vision system assembly for locating and estimating the orientation and size of the target objects for brain-controlled assistive device applications. The inspiration towards the development was the utilization of the intact body function to provide normal grasp manoeuvre to the ALS patients. Often, these functions, considered as high level (cognitive) processes, relied on the users of any grasp assistive device [33,34].

We gave utmost importance to the simplicity and convenience in the realization of the whole system which in turn simplifies the user training. Grasping an object by purely gazing towards it allows the user to concentrate on intentions only rather than on execution. Besides, the proposed image processing pipeline made the system efficient for real-time applications. Due to use of color-based segmentation method, the algorithm can easily avoid the overlapped portions from the segmented object in case of any small color discrepancy between the objects. In future, a colour-independent segmentation method can be developed to avoid mis-identification of the target from its neighbouring object which have the same colour. In this setting, the boundary profile of the objects can identify the orientation of the objects, which not only improves the system’s benefits for clinical use but also reduces the other algorithmic complexity.

In comparison with the existing vision systems [11,12,13,14,15,16], our main objectives for this study were: (1) To develop of a vision system for the users of brain-controlled grasp-assistive device: Most of the existing vision systems [12,15,16] are mostly controlled by the user’s muscle movement; therefore, the neuro-muscular diseased patients are unable to use it. Due to use of gaze variation to control the camera position, the developed system can also be handled by the users’ with muscle impairment. (2) Head-mounted camera position: Most of the available vision system [14,16] are placed on the prosthetic itself; so the prosthetic needs to move towards the target object. Furthermore, it will make the prosthetic heavier in weight which restricts the free movement of it. (3) To avoid additional training for the users: Our developed vision system locate the target object using the gaze movement which is human normal behaviour for object identification. Therefore, no additional training is required for the users. (4) Adopt a simple image processing algorithm to avoid high processing unit: Existing vision systems are using advance machine learning mechanism [12,16,20,21,24] which require high processor and large storage capacity. In the developed vision system, a simple colour based segmentation technique was applied and after that only the object boundary was used for object width and orientation estimation. (5) To avoid any additional sensor(s) for object width estimation: Unlike the other systems, it did not use any sensor for estimation of the object width. We solved the problem with basic trigonometry. (6) To use by the amputee patient also: Our main targeted population is ALS patients, but as the system is controlled by the gaze movement, is can be used by amputee patients also.

Person–object distance measurement is an intrinsic challenge in any vision-based system. Unlike other vision systems [17,18], we did not use any sensor for measuring the distance between the user and the object. Addition of extra hardware will not only make the system complex but also may act as an additional origin for inaccuracies. In the case of smaller objects, the sensor may also miss the object. Furthermore, in a cluttered scene, the sensor may provide with the false result due to interference with other objects. Moreover, it will also add extra cost to the system. Here we approximated the camera to object distance using a simple triangulation method. For this distance calculation, eye level to tabletop distance (*h*) was measured using the normal measuring tape. *h* was assumed to be fixed for the ALS patients as they have very limited control on their voluntary muscle movement. As the gaze angle can provide us with information about the angles, with *h*, we can easily calculate the person–object distance, without adding any extra hardware to the system. As such, we calibrated the system using *h* before starting each experiment. The whole system can work at all reasonable distances between the user and the object.

This study was a proof-of-principle experiment. As such, we did not recruit any ALS patient to take part. Indeed, the performance of the system is independent of the level or the severity of the locked-in state. As long as the patient would be able to move their eyes in both horizontal as well as in vertical direction, they would be able to use the overall system for grasping. In the next stages, we are keen to refine the system in collaboration with potential users. An important issue is the acceptability of this system by users. In the testing phase with able bodied-bodied participants, we also checked their feedback during the use of the device. Due to placement of the device over the participant’s head, they can move their hand freely during the experiment. As they did not require to go through any training program before the experiment, they were feel happy to provide experimental data. During experiment, they were just replicating their normal gazing action to the selected object. However, the present setting requires the user to wear an additional device, that is a goggle so that the EOG signals can be recorded. We used the googles for fixing up the electrode-position; the user can wear it and attach the electrodes at the appropriate place without taking any help from the others. Currently, we are further miniaturizing the EOG system so that it can be applied without the need for glass. It remains to be seen whether removal of the glass could improve the acceptability of the device. Furthermore, in our design, the participant needs to remain fairly fixed in each trial. It remains to be seen whether ALS patients would find this constraint acceptable. Finally, we identified the signatures of objects that are held with Palmar grasp only. We categorized the objects based on their orientation in regular use. We are currently working to include more objects and their signatures under these two and other grasps.

## Data Availability

Data available on request due to privacy of the involved participants.

## Supplementary Materials

The following are available online at https://www.mdpi.com/article/10.3390/s21134515/s1, Video S1: Demo of the EOG controlled vision system.
